# 4-(4-Pyrid­yl)pyridinium bis­(pyridine-2,6-dicarboxyl­ato)ferrate(III) tetra­hydrate

**DOI:** 10.1107/S1600536810008597

**Published:** 2010-03-17

**Authors:** Janet Soleimannejad, Hossein Aghabozorg, Shabnam Sheshmani

**Affiliations:** a Department of Chemistry, Ilam University, Ilam, Iran; b Faculty of Chemistry, Islamic Azad University, North Tehran Branch, Tehran, Iran; c Department of Chemistry, Islamic Azad University, Shahr-e Rey Branch, Tehran, Iran

## Abstract

In the title compound, (C_10_H_9_N_2_)[Fe(C_7_H_3_NO_4_)_2_]·4H_2_O or (bpyH)[Fe(pydc)_2_]·4H_2_O, the asymmetric unit contains an [Fe(pydc)_2_]^−^ (pydcH_2_= pyridine-2,6-dicarboxylic acid) anion, a protonated 4,4′-bipyridine as a counter-ion, (bpyH)^+^, and four uncoordinated water mol­ecules. The anion is a six-coordinate complex with a distorted octa­hedral geometry around the Fe^III ^atom. A wide range of non-covalent inter­actions, *i.e.* O—H⋯O, O—H⋯N and N—H⋯O hydrogen bonds, ion pairing, C—O⋯π [3.431 (2) Å] and C—H⋯π stacking inter­actions result in the formation of a three-dimensional network structure.

## Related literature

For related structures, see: Aghabozorg, Manteghi & Sheshmani (2008 ▶); Aghabozorg, Ramezanipour *et al.* (2008 ▶); Aghajani *et al.* (2009 ▶); For details on the importance of coordinative covalent bonds and weak inter­molecular forces in forming extended organized networks, see: Steiner (2002 ▶).
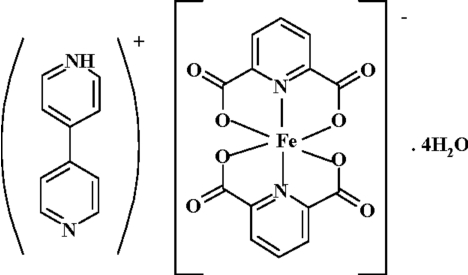


## Experimental

### 

#### Crystal data

(C_10_H_9_N_2_)[Fe(C_7_H_3_NO_4_)_2_]·4H_2_O
*M* *_r_* = 615.31
Triclinic, 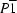

*a* = 9.3759 (9) Å
*b* = 9.3778 (9) Å
*c* = 14.6284 (14) Å
α = 84.545 (2)°
β = 89.246 (2)°
γ = 87.062 (2)°
*V* = 1278.7 (2) Å^3^
*Z* = 2
Mo *K*α radiation
μ = 0.67 mm^−1^
*T* = 150 K
0.39 × 0.39 × 0.28 mm

#### Data collection

Bruker SMART 1000 diffractometer
Absorption correction: multi-scan (*SADABS*; Bruker, 2001 ▶) *T*_min_ = 0.782, *T*_max_ = 0.836
14814 measured reflections
5931 independent reflections
4922 reflections with *I* > 2σ(*I*)
*R*_int_ = 0.030

#### Refinement

*R*[*F* ^2^ > 2σ(*F* ^2^)] = 0.036
*wR*(*F* ^2^) = 0.099
*S* = 1.04
5931 reflections
370 parameters
H-atom parameters constrained
Δρ_max_ = 0.34 e Å^−3^
Δρ_min_ = −0.52 e Å^−3^

### 

Data collection: *SMART* (Bruker, 2007 ▶); cell refinement: *SAINT* (Bruker, 2007 ▶); data reduction: *SAINT*; program(s) used to solve structure: *SHELXS97* (Sheldrick, 2008 ▶); program(s) used to refine structure: *SHELXL97* (Sheldrick, 2008 ▶); molecular graphics: *SHELXTL* (Sheldrick, 2008 ▶) and *Mercury* (Macrae *et al.*, 2006 ▶); software used to prepare material for publication: *SHELXTL*.

## Supplementary Material

Crystal structure: contains datablocks I, global. DOI: 10.1107/S1600536810008597/su2166sup1.cif

Structure factors: contains datablocks I. DOI: 10.1107/S1600536810008597/su2166Isup2.hkl

Additional supplementary materials:  crystallographic information; 3D view; checkCIF report

## Figures and Tables

**Table 1:** Hydrogen-bond geometry (Å, °) *Cg*1 and *Cg*2 are the centroids of the N3/C15–C19 and N1/C2–C6 rings, respectively.

*D*—H⋯*A*	*D*—H	H⋯*A*	*D*⋯*A*	*D*—H⋯*A*
O1*W*—H1*B*⋯O6^i^	0.85	2.22	3.031 (2)	159
O1*W*—H1*A*⋯O3^ii^	0.85	2.18	2.976 (2)	157
O2*W*—H2*B*⋯O7^ii^	0.85	1.93	2.726 (2)	156
O2*W*—H2*A*⋯O3*W*^iii^	0.85	1.88	2.732 (2)	174
O3*W*—H3*A*⋯O5^iv^	0.85	1.90	2.713 (2)	160
O3*W*—H3*B*⋯N3^v^	0.85	1.95	2.775 (2)	162
O4*W*—H4*B*⋯O4	0.85	1.99	2.8220 (19)	168
O4*W*—H4*A*⋯O3*W*	0.85	1.99	2.838 (2)	177
N4—H4*C*⋯O2*W*^vi^	0.90	1.82	2.691 (2)	163
C5—H5⋯*Cg*2	0.95	3.63	3.750 (2)	90
C17—H17⋯*Cg*1	0.95	3.52	3.708 (2)	94

Symmetry codes: (i) 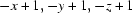; (ii) 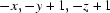; (iii) 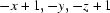; (iv) 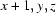; (v) 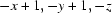; (vi) 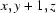.

**Fig. 1.**
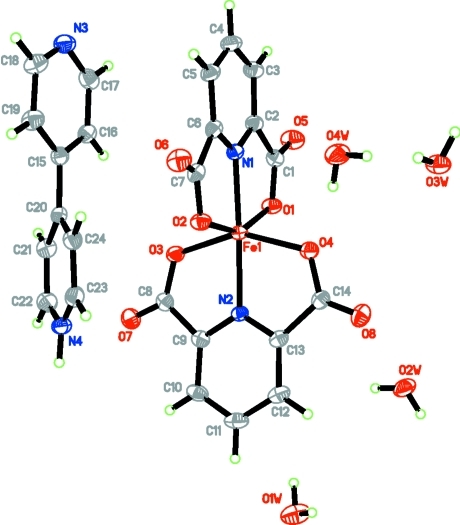
Molecular structure of the title compound, (bpyH)[Fe(pydc)2].4H2O. Thermal ellipsoids are drawn at the 50% probability level.

**Fig. 2.**
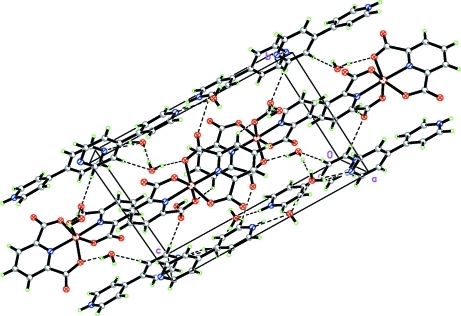
A perspective view, along the a-axis, of the crystal packing of the title compound [dashed lines indicate hydrogen bonds - see Table 1 for details].

**Fig. 3.**
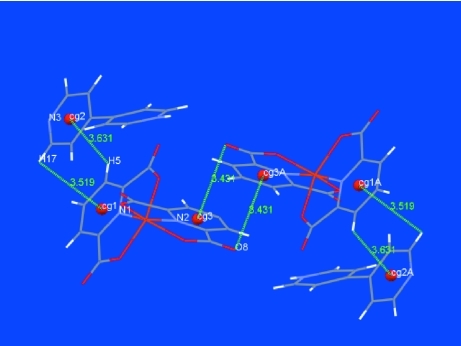
A view of the C–O···π and C–H···π interactions in the title compound. The H17···Cg1 (Cg1: centroid of ring N1, C2—C6) distance is 3.519 (2) Å, the H5···Cg2 (Cg2: centroid of ring N3, C15—C19) distance is 3.631 (2) Å and the O8···Cg3 A (Cg3 A: centroid of ring N2A, C9A—C13A) distance is 3.431 (2) Å [Symmetry code: (A) = –x, –y, –z].

## supplementary crystallographic information

### Comment

For the synthesis of supramolecular systems, coordinative covalent bonds and weak intermolecular forces are important to the assembly into extended organized networks (Steiner,  2002). Our research group has worked on the synthesis of supramolecular systems, and found out the role of non-covalent interactions such as hydrogen bonding, ion pairing and π-π stacking in constructing the supramolecular crystalline compounds and their metal complexes (Aghabozorg, Manteghi *et al.*, 2008; Aghabozorg, Ramezanipour *et al.*, 2008, Aghajani *et al.*, 2009).

In the title compound, illustrated in Fig. 1, the metal ion is hexa-coordinated by two nitrogen atoms N1, and N2 and four oxygen atoms O1, O2, O3 and O4 of carboxylate groups of two (pydc)^2–^ ions. The Fe^III^ atom is located in the center of a distorted octahedral arrangement. The N1–Fe–N2 angle shows deviation from linearity, 170.90 (6)°. The O2–Fe1–O4–C14, O2–Fe1–O3–C8, O4–Fe1–O1–C1 and O4–Fe1–O2–C7 torsion angles are 86.75 (14)°, -93.74 (13)°, -102.35 (14)° and 94.37 (14)°, respectively, indicating that two dianionic (pydc)^2–^ units are almost perpendicular to each other. Another characteristic solid state structural feature of this complex is dictated by the presence of a 4,4'-bipyridinium fragment as a proton acceptor that deprotonates pyridine-2,6-dicarboxylic acid. This leads to the formation of a metal-organo Fe^III^ complex in which ion-pairing, metal-ligand coordination and intensive hydrogen-bonding play important roles in the construction of its three dimensional supramolecular network.

The crystal packing diagram (Fig. 2) indicates the interesting layered structure for the title complex. The space provided between two layers, consisting of (bpyH)^+^ cations, are filled with a layer of [Fe(pydc)_2_]^–^ complex anions. In fact, the layers involving the Fe^III^complexes are bridged by (bpyH)^+^ counter ions *via* hydrogen bonding. The hydrogen bonding, that is O–H···O, O–H···N and N–H···O between carboxylate, (bpyH)^+^ and water molecules, throughout the lattice of the title complex plays an important role in stabilizing the crystal structure (Fig 2 and Table 1).

The C–O···π and C–H···π interactions in the title compound are shown in Fig. 3. The H17···*Cg*1 (*Cg*1: N1, C2—C6) distance is 3.519 (2) Å, the H5···*Cg*2 (*Cg*2: N3, C15—C19) distance is 3.631 (2) Å and the O8···*Cg*3 A (*Cg*3 A: N2A, C9A—C13A) distance is 3.431 (2) Å [Symmetry code: (A) = –*x*, –*y*, –*z*].

### Experimental

An solution of pyridine-2,6-dicarboxylic acid (312.38 mg, 2 mmol) and 4,4'-bipyridine (167.12 mg, 1 mmol) in water (15 ml) was refluxed for 1h. To this mixture was added to a solution of FeCl_2_.4H_2_O (99.4 mg, 0.5 mmol) in water (5 ml) and it was then heated for a further 1h. Green crystals of the complex, suitable for X-ray analysis, were obtained by slow evaporation of the solution at RT after two weeks.

### Refinement

The water and NH H-atoms were located in a low 2θ difference Fourier map and refined with distance restraints: O—H = 0.85 (2) Å, and N-H 0.89 (2) Å. The C-bound H-atoms were included in calculated positions and treated as riding atoms: C—H = 0.95 Å. For all H-atoms *U*_iso_(H) = 1.2*U*_eq_(parent O-, N- or C-atom).

### Crystal data

**Table:**

(C_10_H_9_N_2_)[Fe(C_7_H_3_NO_4_)_2_]·4H_2_O	*Z* = 2
*M**_r_* = 615.31	*F*(000) = 634
Triclinic, *P*1	*D*_x_ = 1.598 Mg m^−^^3^
Hall symbol: -P 1	Mo *K*α radiation, λ = 0.71073 Å
*a* = 9.3759 (9) Å	Cell parameters from 7438 reflections
*b* = 9.3778 (9) Å	θ = 2.5–28.6°
*c* = 14.6284 (14) Å	µ = 0.67 mm^−^^1^
α = 84.545 (2)°	*T* = 150 K
β = 89.246 (2)°	Block, green
γ = 87.062 (2)°	0.39 × 0.39 × 0.28 mm
*V* = 1278.7 (2) Å^3^	

### Data collection

**Table:**

Bruker SMART 1000 diffractometer	5931 independent reflections
Radiation source: fine-focus sealed tube	4922 reflections with *I* > 2σ(*I*)
graphite	*R*_int_ = 0.030
Detector resolution: 100 pixels mm^-1^	θ_max_ = 28.7°, θ_min_ = 1.4°
ω scans	*h* = −12→11
Absorption correction: multi-scan (*SADABS*; Bruker, 2001)	*k* = −12→12
*T*_min_ = 0.782, *T*_max_ = 0.836	*l* = −19→19
14814 measured reflections	

### Refinement

**Table:**

Refinement on *F*^2^	Primary atom site location: structure-invariant direct methods
Least-squares matrix: full	Secondary atom site location: difference Fourier map
*R*[*F*^2^ > 2σ(*F*^2^)] = 0.036	Hydrogen site location: inferred from neighbouring sites
*wR*(*F*^2^) = 0.099	H-atom parameters constrained
*S* = 1.04	*w* = 1/[σ^2^(*F*_o_^2^) + (0.0563*P*)^2^ + 0.2243*P*] where *P* = (*F*_o_^2^ + 2*F*_c_^2^)/3
5931 reflections	(Δ/σ)_max_ = 0.001
370 parameters	Δρ_max_ = 0.34 e Å^−^^3^
0 restraints	Δρ_min_ = −0.51 e Å^−^^3^

### Special details

**Table:**

Geometry. All esds (except the esd in the dihedral angle between two l.s. planes) are estimated using the full covariance matrix. The cell esds are taken into account individually in the estimation of esds in distances, angles and torsion angles; correlations between esds in cell parameters are only used when they are defined by crystal symmetry. An approximate (isotropic) treatment of cell esds is used for estimating esds involving l.s. planes.
Refinement. Refinement of *F*^2^ against ALL reflections. The weighted *R*-factor wR and goodness of fit *S* are based on *F*^2^, conventional *R*-factors *R* are based on *F*, with *F* set to zero for negative *F*^2^. The threshold expression of *F*^2^ > σ(*F*^2^) is used only for calculating *R*-factors(gt) etc. and is not relevant to the choice of reflections for refinement. *R*-factors based on *F*^2^ are statistically about twice as large as those based on *F*, and *R*- factors based on ALL data will be even larger.

### Fractional atomic coordinates and isotropic or equivalent isotropic displacement parameters (Å^2^)

**Table:**

	*x*	*y*	*z*	*U*_iso_*/*U*_eq_	
Fe1	0.22857 (3)	0.50271 (3)	0.304811 (17)	0.01665 (9)	
O1W	0.12803 (17)	0.32200 (19)	0.82149 (11)	0.0425 (4)	
H1B	0.2145	0.2957	0.8116	0.051*	
H1A	0.0913	0.3184	0.7690	0.051*	
O1	0.07928 (14)	0.37678 (14)	0.26484 (8)	0.0216 (3)	
O2W	0.25021 (16)	0.03484 (17)	0.60895 (9)	0.0356 (4)	
H2B	0.1849	0.1012	0.6087	0.043*	
H2A	0.2416	−0.0150	0.6602	0.043*	
O2	0.38786 (13)	0.63723 (14)	0.27794 (9)	0.0212 (3)	
O3	0.07590 (14)	0.65525 (14)	0.33404 (9)	0.0213 (3)	
O3W	0.77869 (15)	0.10812 (15)	0.22072 (9)	0.0273 (3)	
H3A	0.8334	0.1769	0.2080	0.033*	
H3B	0.7778	0.0637	0.1727	0.033*	
O4	0.36699 (14)	0.33767 (14)	0.34487 (9)	0.0223 (3)	
O4W	0.53959 (16)	0.30365 (17)	0.18817 (10)	0.0342 (4)	
H4B	0.4969	0.3050	0.2397	0.041*	
H4A	0.6119	0.2454	0.1960	0.041*	
O5	−0.02554 (15)	0.29003 (16)	0.14699 (10)	0.0308 (3)	
O6	0.54940 (14)	0.72094 (15)	0.17475 (10)	0.0274 (3)	
O7	−0.03745 (14)	0.76723 (15)	0.44505 (10)	0.0280 (3)	
O8	0.50477 (15)	0.23244 (15)	0.45845 (10)	0.0277 (3)	
N1	0.25170 (15)	0.51693 (16)	0.16448 (10)	0.0171 (3)	
N2	0.23620 (15)	0.50323 (16)	0.44512 (10)	0.0166 (3)	
N3	0.22726 (18)	0.97703 (19)	−0.04433 (11)	0.0269 (4)	
N4	0.28135 (18)	0.99456 (17)	0.42996 (11)	0.0244 (4)	
H4C	0.2889	1.0021	0.4902	0.029*	
C1	0.06258 (19)	0.3626 (2)	0.17921 (13)	0.0210 (4)	
C2	0.16527 (19)	0.4448 (2)	0.11620 (12)	0.0194 (4)	
C3	0.1780 (2)	0.4493 (2)	0.02214 (13)	0.0233 (4)	
H3	0.1173	0.3968	−0.0123	0.028*	
C4	0.2818 (2)	0.5326 (2)	−0.02104 (13)	0.0248 (4)	
H4	0.2916	0.5391	−0.0860	0.030*	
C5	0.3719 (2)	0.6067 (2)	0.03041 (13)	0.0219 (4)	
H5	0.4434	0.6638	0.0015	0.026*	
C6	0.35415 (19)	0.59466 (19)	0.12467 (13)	0.0187 (4)	
C7	0.44092 (19)	0.65836 (19)	0.19604 (13)	0.0200 (4)	
C8	0.05410 (19)	0.6827 (2)	0.41791 (13)	0.0206 (4)	
C9	0.15317 (19)	0.59809 (19)	0.48584 (12)	0.0192 (4)	
C10	0.1634 (2)	0.6097 (2)	0.57899 (13)	0.0237 (4)	
H10	0.1047	0.6778	0.6083	0.028*	
C11	0.2620 (2)	0.5190 (2)	0.62818 (13)	0.0268 (4)	
H11	0.2717	0.5251	0.6922	0.032*	
C12	0.3471 (2)	0.4188 (2)	0.58516 (13)	0.0234 (4)	
H12	0.4142	0.3557	0.6188	0.028*	
C13	0.33042 (18)	0.41466 (19)	0.49149 (12)	0.0183 (4)	
C14	0.41062 (19)	0.3178 (2)	0.42920 (13)	0.0196 (4)	
C15	0.2438 (2)	0.9931 (2)	0.14461 (13)	0.0205 (4)	
C16	0.1444 (2)	0.9092 (2)	0.10825 (13)	0.0223 (4)	
H16	0.0793	0.8574	0.1472	0.027*	
C17	0.1425 (2)	0.9031 (2)	0.01440 (14)	0.0264 (4)	
H17	0.0769	0.8427	−0.0097	0.032*	
C18	0.3193 (2)	1.0607 (2)	−0.00902 (14)	0.0296 (5)	
H18	0.3784	1.1164	−0.0501	0.036*	
C19	0.3330 (2)	1.0707 (2)	0.08356 (14)	0.0266 (4)	
H19	0.4021	1.1294	0.1056	0.032*	
C20	0.25691 (19)	0.99744 (19)	0.24448 (12)	0.0190 (4)	
C21	0.1369 (2)	1.0210 (2)	0.29944 (13)	0.0232 (4)	
H21	0.0449	1.0364	0.2727	0.028*	
C22	0.1529 (2)	1.0217 (2)	0.39210 (14)	0.0258 (4)	
H22	0.0723	1.0417	0.4297	0.031*	
C23	0.3993 (2)	0.9715 (2)	0.37953 (13)	0.0241 (4)	
H23	0.4892	0.9528	0.4086	0.029*	
C24	0.3899 (2)	0.9751 (2)	0.28632 (13)	0.0220 (4)	
H24	0.4736	0.9623	0.2501	0.026*	

### Atomic displacement parameters (Å^2^)

**Table:**

	*U*^11^	*U*^22^	*U*^33^	*U*^12^	*U*^13^	*U*^23^
Fe1	0.01791 (14)	0.01966 (15)	0.01226 (14)	−0.00143 (10)	0.00009 (10)	−0.00068 (10)
O1W	0.0339 (9)	0.0640 (12)	0.0285 (8)	−0.0033 (8)	−0.0037 (7)	0.0012 (8)
O1	0.0220 (7)	0.0266 (7)	0.0167 (6)	−0.0064 (5)	0.0013 (5)	−0.0019 (5)
O2W	0.0389 (9)	0.0457 (9)	0.0185 (7)	0.0174 (7)	0.0055 (6)	0.0049 (6)
O2	0.0222 (7)	0.0256 (7)	0.0165 (6)	−0.0060 (5)	−0.0016 (5)	−0.0021 (5)
O3	0.0222 (7)	0.0237 (7)	0.0174 (6)	0.0022 (5)	−0.0021 (5)	−0.0011 (5)
O3W	0.0310 (8)	0.0322 (8)	0.0191 (7)	−0.0054 (6)	0.0032 (6)	−0.0027 (6)
O4	0.0248 (7)	0.0237 (7)	0.0179 (7)	0.0030 (5)	0.0004 (5)	−0.0018 (5)
O4W	0.0325 (8)	0.0423 (9)	0.0255 (8)	0.0069 (7)	0.0086 (6)	0.0026 (6)
O5	0.0305 (8)	0.0375 (8)	0.0265 (8)	−0.0144 (6)	−0.0019 (6)	−0.0058 (6)
O6	0.0230 (7)	0.0316 (8)	0.0279 (8)	−0.0095 (6)	0.0022 (6)	−0.0002 (6)
O7	0.0256 (7)	0.0281 (8)	0.0298 (8)	0.0052 (6)	0.0029 (6)	−0.0048 (6)
O8	0.0254 (7)	0.0257 (7)	0.0306 (8)	0.0041 (6)	−0.0030 (6)	0.0024 (6)
N1	0.0168 (7)	0.0186 (7)	0.0158 (7)	−0.0015 (6)	0.0006 (6)	−0.0001 (6)
N2	0.0174 (7)	0.0184 (7)	0.0140 (7)	−0.0032 (6)	0.0005 (6)	0.0001 (6)
N3	0.0287 (9)	0.0309 (9)	0.0205 (8)	0.0030 (7)	−0.0010 (7)	−0.0022 (7)
N4	0.0338 (9)	0.0233 (8)	0.0164 (8)	−0.0067 (7)	−0.0013 (7)	−0.0009 (6)
C1	0.0198 (9)	0.0224 (9)	0.0210 (9)	−0.0013 (7)	−0.0003 (7)	−0.0033 (7)
C2	0.0185 (9)	0.0224 (9)	0.0174 (9)	0.0002 (7)	−0.0017 (7)	−0.0026 (7)
C3	0.0227 (9)	0.0288 (10)	0.0190 (9)	−0.0006 (8)	−0.0024 (8)	−0.0044 (8)
C4	0.0267 (10)	0.0314 (11)	0.0152 (9)	0.0033 (8)	0.0017 (8)	0.0004 (8)
C5	0.0215 (9)	0.0245 (10)	0.0183 (9)	0.0014 (7)	0.0033 (7)	0.0027 (7)
C6	0.0164 (8)	0.0191 (9)	0.0197 (9)	0.0011 (7)	0.0015 (7)	0.0009 (7)
C7	0.0190 (9)	0.0189 (9)	0.0213 (9)	0.0002 (7)	−0.0011 (7)	0.0019 (7)
C8	0.0190 (9)	0.0204 (9)	0.0230 (10)	−0.0049 (7)	0.0015 (7)	−0.0030 (7)
C9	0.0182 (9)	0.0213 (9)	0.0187 (9)	−0.0053 (7)	0.0024 (7)	−0.0028 (7)
C10	0.0242 (10)	0.0295 (11)	0.0185 (9)	−0.0076 (8)	0.0057 (8)	−0.0062 (8)
C11	0.0309 (11)	0.0357 (11)	0.0149 (9)	−0.0117 (9)	0.0008 (8)	−0.0025 (8)
C12	0.0225 (9)	0.0290 (10)	0.0184 (9)	−0.0067 (8)	−0.0034 (7)	0.0033 (8)
C13	0.0155 (8)	0.0216 (9)	0.0174 (9)	−0.0050 (7)	0.0005 (7)	0.0017 (7)
C14	0.0182 (9)	0.0192 (9)	0.0210 (9)	−0.0042 (7)	0.0005 (7)	0.0018 (7)
C15	0.0207 (9)	0.0203 (9)	0.0201 (9)	0.0013 (7)	0.0002 (7)	−0.0015 (7)
C16	0.0217 (9)	0.0223 (9)	0.0224 (10)	−0.0020 (7)	0.0004 (8)	0.0001 (7)
C17	0.0252 (10)	0.0276 (10)	0.0271 (10)	0.0016 (8)	−0.0059 (8)	−0.0072 (8)
C18	0.0301 (11)	0.0349 (12)	0.0230 (10)	−0.0050 (9)	0.0025 (8)	0.0030 (8)
C19	0.0250 (10)	0.0301 (11)	0.0249 (10)	−0.0070 (8)	0.0001 (8)	−0.0002 (8)
C20	0.0227 (9)	0.0152 (9)	0.0189 (9)	−0.0023 (7)	−0.0006 (7)	0.0000 (7)
C21	0.0196 (9)	0.0258 (10)	0.0241 (10)	−0.0019 (8)	0.0005 (8)	−0.0018 (8)
C22	0.0264 (10)	0.0263 (10)	0.0249 (10)	−0.0054 (8)	0.0062 (8)	−0.0024 (8)
C23	0.0257 (10)	0.0204 (9)	0.0260 (10)	−0.0024 (8)	−0.0050 (8)	−0.0002 (8)
C24	0.0219 (9)	0.0208 (9)	0.0234 (10)	−0.0010 (7)	0.0012 (8)	−0.0028 (7)

### Geometric parameters (Å, °)

**Table:**

Fe1—O1	2.0045 (13)	C3—C4	1.388 (3)
Fe1—O2	2.0149 (13)	C3—H3	0.9500
Fe1—O4	2.0161 (13)	C4—C5	1.392 (3)
Fe1—O3	2.0417 (13)	C4—H4	0.9500
Fe1—N1	2.0538 (15)	C5—C6	1.381 (3)
Fe1—N2	2.0552 (15)	C5—H5	0.9500
O1W—H1B	0.8500	C6—C7	1.516 (3)
O1W—H1A	0.8501	C8—C9	1.510 (3)
O1—C1	1.285 (2)	C9—C10	1.382 (3)
O2W—H2B	0.8500	C10—C11	1.385 (3)
O2W—H2A	0.8500	C10—H10	0.9500
O2—C7	1.293 (2)	C11—C12	1.392 (3)
O3—C8	1.289 (2)	C11—H11	0.9500
O3W—H3A	0.8500	C12—C13	1.386 (3)
O3W—H3B	0.8501	C12—H12	0.9500
O4—C14	1.298 (2)	C13—C14	1.513 (3)
O4W—H4B	0.8500	C15—C16	1.392 (3)
O4W—H4A	0.8500	C15—C19	1.395 (3)
O5—C1	1.224 (2)	C15—C20	1.472 (3)
O6—C7	1.222 (2)	C16—C17	1.380 (3)
O7—C8	1.226 (2)	C16—H16	0.9500
O8—C14	1.215 (2)	C17—H17	0.9500
N1—C6	1.330 (2)	C18—C19	1.374 (3)
N1—C2	1.332 (2)	C18—H18	0.9500
N2—C13	1.328 (2)	C19—H19	0.9500
N2—C9	1.330 (2)	C20—C24	1.393 (3)
N3—C17	1.333 (3)	C20—C21	1.395 (3)
N3—C18	1.337 (3)	C21—C22	1.366 (3)
N4—C22	1.335 (3)	C21—H21	0.9500
N4—C23	1.341 (3)	C22—H22	0.9500
N4—H4C	0.8951	C23—C24	1.365 (3)
C1—C2	1.513 (3)	C23—H23	0.9500
C2—C3	1.376 (3)	C24—H24	0.9500
			
O1—Fe1—O2	151.86 (5)	O2—C7—C6	112.77 (15)
O1—Fe1—O4	94.25 (5)	O7—C8—O3	126.24 (18)
O2—Fe1—O4	92.07 (5)	O7—C8—C9	119.82 (17)
O1—Fe1—O3	91.06 (5)	O3—C8—C9	113.94 (15)
O2—Fe1—O3	96.57 (5)	N2—C9—C10	120.84 (18)
O4—Fe1—O3	150.97 (5)	N2—C9—C8	111.22 (15)
O1—Fe1—N1	76.29 (5)	C10—C9—C8	127.94 (17)
O2—Fe1—N1	75.58 (5)	C9—C10—C11	117.76 (18)
O4—Fe1—N1	101.99 (6)	C9—C10—H10	121.1
O3—Fe1—N1	106.99 (5)	C11—C10—H10	121.1
O1—Fe1—N2	112.55 (5)	C10—C11—C12	120.97 (18)
O2—Fe1—N2	95.58 (5)	C10—C11—H11	119.5
O4—Fe1—N2	75.80 (6)	C12—C11—H11	119.5
O3—Fe1—N2	75.81 (5)	C13—C12—C11	117.54 (18)
N1—Fe1—N2	170.90 (6)	C13—C12—H12	121.2
H1B—O1W—H1A	101.7	C11—C12—H12	121.2
C1—O1—Fe1	120.32 (12)	N2—C13—C12	120.78 (17)
H2B—O2W—H2A	105.6	N2—C13—C14	111.52 (15)
C7—O2—Fe1	120.81 (11)	C12—C13—C14	127.69 (17)
C8—O3—Fe1	119.73 (12)	O8—C14—O4	126.06 (18)
H3A—O3W—H3B	105.5	O8—C14—C13	121.30 (17)
C14—O4—Fe1	120.54 (12)	O4—C14—C13	112.64 (16)
H4B—O4W—H4A	107.2	C16—C15—C19	117.95 (18)
C6—N1—C2	121.95 (16)	C16—C15—C20	121.17 (17)
C6—N1—Fe1	119.33 (12)	C19—C15—C20	120.87 (17)
C2—N1—Fe1	118.70 (12)	C17—C16—C15	118.55 (18)
C13—N2—C9	122.10 (16)	C17—C16—H16	120.7
C13—N2—Fe1	118.71 (12)	C15—C16—H16	120.7
C9—N2—Fe1	119.05 (12)	N3—C17—C16	123.77 (18)
C17—N3—C18	117.23 (17)	N3—C17—H17	118.1
C22—N4—C23	122.20 (17)	C16—C17—H17	118.1
C22—N4—H4C	117.7	N3—C18—C19	123.52 (19)
C23—N4—H4C	119.7	N3—C18—H18	118.2
O5—C1—O1	125.95 (18)	C19—C18—H18	118.2
O5—C1—C2	119.91 (17)	C18—C19—C15	118.90 (19)
O1—C1—C2	114.13 (15)	C18—C19—H19	120.5
N1—C2—C3	120.86 (17)	C15—C19—H19	120.5
N1—C2—C1	110.55 (15)	C24—C20—C21	118.58 (17)
C3—C2—C1	128.58 (17)	C24—C20—C15	120.40 (17)
C2—C3—C4	118.15 (17)	C21—C20—C15	121.00 (17)
C2—C3—H3	120.9	C22—C21—C20	119.35 (18)
C4—C3—H3	120.9	C22—C21—H21	120.3
C3—C4—C5	120.30 (17)	C20—C21—H21	120.3
C3—C4—H4	119.9	N4—C22—C21	120.22 (18)
C5—C4—H4	119.9	N4—C22—H22	119.9
C6—C5—C4	118.09 (17)	C21—C22—H22	119.9
C6—C5—H5	121.0	N4—C23—C24	119.81 (18)
C4—C5—H5	121.0	N4—C23—H23	120.1
N1—C6—C5	120.63 (17)	C24—C23—H23	120.1
N1—C6—C7	110.76 (15)	C23—C24—C20	119.74 (18)
C5—C6—C7	128.61 (17)	C23—C24—H24	120.1
O6—C7—O2	126.05 (17)	C20—C24—H24	120.1
O6—C7—C6	121.18 (17)		
			
O2—Fe1—O1—C1	0.1 (2)	C4—C5—C6—N1	−1.5 (3)
O4—Fe1—O1—C1	−102.35 (14)	C4—C5—C6—C7	177.18 (18)
O3—Fe1—O1—C1	106.21 (14)	Fe1—O2—C7—O6	−169.14 (15)
N1—Fe1—O1—C1	−1.03 (13)	Fe1—O2—C7—C6	10.3 (2)
N2—Fe1—O1—C1	−178.73 (13)	N1—C6—C7—O6	172.26 (17)
O1—Fe1—O2—C7	−8.6 (2)	C5—C6—C7—O6	−6.5 (3)
O4—Fe1—O2—C7	94.37 (14)	N1—C6—C7—O2	−7.2 (2)
O3—Fe1—O2—C7	−113.39 (14)	C5—C6—C7—O2	174.08 (18)
N1—Fe1—O2—C7	−7.48 (13)	Fe1—O3—C8—O7	−176.98 (15)
N2—Fe1—O2—C7	170.30 (13)	Fe1—O3—C8—C9	2.3 (2)
O1—Fe1—O3—C8	113.40 (13)	C13—N2—C9—C10	1.0 (3)
O2—Fe1—O3—C8	−93.74 (13)	Fe1—N2—C9—C10	−174.73 (13)
O4—Fe1—O3—C8	12.7 (2)	C13—N2—C9—C8	−178.78 (15)
N1—Fe1—O3—C8	−170.62 (13)	Fe1—N2—C9—C8	5.50 (19)
N2—Fe1—O3—C8	0.39 (13)	O7—C8—C9—N2	174.39 (17)
O1—Fe1—O4—C14	−120.69 (13)	O3—C8—C9—N2	−4.9 (2)
O2—Fe1—O4—C14	86.75 (14)	O7—C8—C9—C10	−5.4 (3)
O3—Fe1—O4—C14	−20.8 (2)	O3—C8—C9—C10	175.31 (17)
N1—Fe1—O4—C14	162.44 (13)	N2—C9—C10—C11	−0.4 (3)
N2—Fe1—O4—C14	−8.49 (13)	C8—C9—C10—C11	179.32 (17)
O1—Fe1—N1—C6	−177.84 (14)	C9—C10—C11—C12	−0.4 (3)
O2—Fe1—N1—C6	2.71 (13)	C10—C11—C12—C13	0.7 (3)
O4—Fe1—N1—C6	−86.34 (14)	C9—N2—C13—C12	−0.7 (3)
O3—Fe1—N1—C6	95.29 (14)	Fe1—N2—C13—C12	175.02 (13)
O1—Fe1—N1—C2	0.59 (13)	C9—N2—C13—C14	179.86 (15)
O2—Fe1—N1—C2	−178.86 (14)	Fe1—N2—C13—C14	−4.41 (19)
O4—Fe1—N1—C2	92.09 (14)	C11—C12—C13—N2	−0.1 (3)
O3—Fe1—N1—C2	−86.28 (14)	C11—C12—C13—C14	179.21 (17)
O1—Fe1—N2—C13	95.46 (13)	Fe1—O4—C14—O8	−171.42 (15)
O2—Fe1—N2—C13	−83.99 (13)	Fe1—O4—C14—C13	8.6 (2)
O4—Fe1—N2—C13	6.73 (12)	N2—C13—C14—O8	177.55 (16)
O3—Fe1—N2—C13	−179.38 (14)	C12—C13—C14—O8	−1.8 (3)
O1—Fe1—N2—C9	−88.68 (14)	N2—C13—C14—O4	−2.5 (2)
O2—Fe1—N2—C9	91.87 (13)	C12—C13—C14—O4	178.16 (17)
O4—Fe1—N2—C9	−177.40 (14)	C19—C15—C16—C17	2.3 (3)
O3—Fe1—N2—C9	−3.51 (13)	C20—C15—C16—C17	−176.39 (17)
Fe1—O1—C1—O5	−179.29 (15)	C18—N3—C17—C16	0.4 (3)
Fe1—O1—C1—C2	1.2 (2)	C15—C16—C17—N3	−2.5 (3)
C6—N1—C2—C3	−0.7 (3)	C17—N3—C18—C19	1.9 (3)
Fe1—N1—C2—C3	−179.05 (14)	N3—C18—C19—C15	−2.0 (3)
C6—N1—C2—C1	178.22 (16)	C16—C15—C19—C18	−0.2 (3)
Fe1—N1—C2—C1	−0.2 (2)	C20—C15—C19—C18	178.46 (18)
O5—C1—C2—N1	179.83 (17)	C16—C15—C20—C24	129.3 (2)
O1—C1—C2—N1	−0.7 (2)	C19—C15—C20—C24	−49.3 (3)
O5—C1—C2—C3	−1.4 (3)	C16—C15—C20—C21	−49.4 (3)
O1—C1—C2—C3	178.12 (18)	C19—C15—C20—C21	132.0 (2)
N1—C2—C3—C4	−0.9 (3)	C24—C20—C21—C22	0.1 (3)
C1—C2—C3—C4	−179.59 (18)	C15—C20—C21—C22	178.81 (17)
C2—C3—C4—C5	1.3 (3)	C23—N4—C22—C21	2.8 (3)
C3—C4—C5—C6	−0.1 (3)	C20—C21—C22—N4	−2.6 (3)
C2—N1—C6—C5	1.9 (3)	C22—N4—C23—C24	−0.2 (3)
Fe1—N1—C6—C5	−179.73 (13)	N4—C23—C24—C20	−2.3 (3)
C2—N1—C6—C7	−176.97 (16)	C21—C20—C24—C23	2.3 (3)
Fe1—N1—C6—C7	1.40 (19)	C15—C20—C24—C23	−176.38 (17)

### Hydrogen-bond geometry (Å, °)

**Table:**

Cg1 and Cg2 are the centroids of the N3/C15–C19 and N1/C2–C6 rings, respectively.

**Table:**

*D*—H···*A*	*D*—H	H···*A*	*D*···*A*	*D*—H···*A*
O1W—H1B···O6^i^	0.85	2.22	3.031 (2)	159
O1W—H1A···O3^ii^	0.85	2.18	2.976 (2)	157
O2W—H2B···O7^ii^	0.85	1.93	2.726 (2)	156
O2W—H2A···O3W^iii^	0.85	1.88	2.732 (2)	174
O3W—H3A···O5^iv^	0.85	1.90	2.713 (2)	160
O3W—H3B···N3^v^	0.85	1.95	2.775 (2)	162
O4W—H4B···O4	0.85	1.99	2.8220 (19)	168
O4W—H4A···O3W	0.85	1.99	2.838 (2)	177
N4—H4C···O2W^vi^	0.90	1.82	2.691 (2)	163
C5—H5···Cg2	0.95	3.63	3.750 (2)	90
C17—H17···Cg1	0.95	3.52	3.708 (2)	94

Symmetry codes: (i) −*x*+1, −*y*+1, −*z*+1; (ii) −*x*, −*y*+1, −*z*+1; (iii) −*x*+1, −*y*, −*z*+1; (iv) *x*+1, *y*, *z*; (v) −*x*+1, −*y*+1, −*z*; (vi) *x*, *y*+1, *z*.
